# Secondary Metabolites from the Deep-Sea Derived Fungus *Acaromyces ingoldii* FS121

**DOI:** 10.3390/molecules21040371

**Published:** 2016-03-29

**Authors:** Xiao-Wei Gao, Hong-Xin Liu, Zhang-Hua Sun, Yu-Chan Chen, Yu-Zhi Tan, Wei-Min Zhang

**Affiliations:** 1 State Key Laboratory of Applied Microbiology Southern China, Guangdong Provincial Key Laboratory of Microbial Culture Collection and Application, Guangdong Open Laboratory of Applied Microbiology, Guangdong Institute of Microbiology, Guangzhou 510070, China; xiao.wei96@163.com (X.-W.G.); hxinliu1225@163.com (H.-X.L.); sysuszh@126.com (Z.-H.S.); yuchan2006@126.com (Y.-C.C.); 2 College of Pharmacy, Guangdong Pharmaceutical University, Guangzhou 510006, China; tanyuzhi@163.com

**Keywords:** *Acaromyces ingoldii*, secondary metabolites, deep-sea derived fungus, cell growth inhibition

## Abstract

Activity-guided isolation of the fermentation broth of the deep-sea derived fungus *Acaromyces ingoldii* FS121, which was obtained from the China South Sea, yielded a new naphtha-[2,3-*b*]pyrandione analogue, acaromycin A (**1**) and a new thiazole analogue, acaromyester A (**2**), as well as the known compound (+)-cryptosporin (**3**). Their structures, including absolute configurations, were determined by extensive spectroscopic analysis and electronic circular dichroism (ECD) spectra. Compounds **1**–**3** were evaluated for *in vitro* growth inhibitory activities against four tumor cell lines (MCF-7, NCI-H460, SF-268 and HepG-2), wherein compounds **1** and **3** exhibited considerable growth inhibitory effects, with IC_50_ values less than 10 µM.

## 1. Introduction

Marine organisms live in a biologically competitive environment with unique environmental conditions, therefore marine-derived fungi have been proven to be a rich source of structurally unique and biologically active secondary metabolites [1,2,3,4,5,6,7,8,9,10]. In spite of the fact that the investigations of fungal metabolites and their derivatives have not yet led to a clinical cancer drug, significant research efforts have revealed a large number of fungi-derived natural products with promising anticancer activity [11,12,13]. Such chemical entities not only provide insight into the biogenetic landscape, revealing new structure classes and new biosynthetic pathways, but also enlighten the approach to biomimetic synthesis and further bioactivity investigations, suggesting potential candidates for the development of new pharmaceutical agents [14].

**Figure 1:**
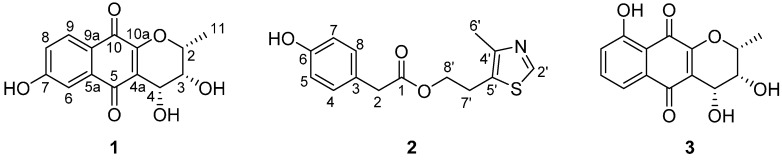
Chemical structures of compounds **1**–**3**.

In the course of our search for new bioactive natural products from marine-derived fungi, we found that a culture broth of *Acaromyces ingoldii* FS121 showed potent cell growth inhibitory effects against SF-268, MCF-7, NCI-H460 and HepG-2 human tumor cell lines [15]. Further chemical investigation of this strain led to the isolation of two new secondary metabolites—acaromycin A (**1**) and acaromyester A (**2**)—along with the known compound (+)-cryptosporin (**3**) (Figure 1). Herein, details of the isolation, structure elucidation, and cell growth inhibition effects of these compounds are described.

## 2. Results

### 2.1. Structural Elucidation of New Compounds

**Figure 2:**
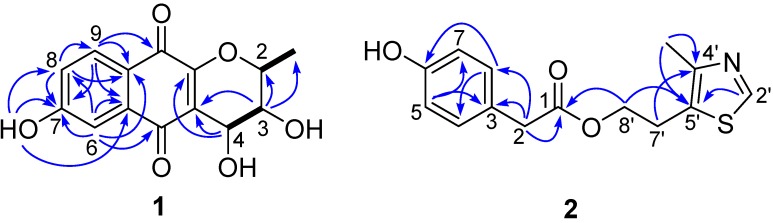
^1^H, ^1^H-COSY and key HMBCs of **1** and **2**.

Acaromycin A (**1**) was isolated as an orange amorphous powder with the molecular formula of C_14_H_12_O_6_ as determined by HRESIMS (*m*/*z* 299.0533, [M + Na]^+^, calcd. for 299.0532), corresponding to nine degrees of unsaturation. The IR spectrum exhibited absorption bands for OH (3309 cm^−1^) and carbonyl (1647 and 1625 cm^−1^) groups. The ^1^H-NMR data (Table 1) exhibited signals for a methyl proton δ_H_ 1.65 (H_3_-11, d, *J* = 6.7 Hz), three aromatic protons δ_H_ 7.61 (H-9, d, *J* = 1.9 Hz), 7.60 (H-6, s), 7.23 (H-8, dd, *J* = 5.6, 4.0 Hz), three methine protons δ_H_ 4.96 (H-4, d, *J* = 4.3, 0.9 Hz), 4.30 (H-2, q, *J* = 6.7, 1.0 Hz), 4.03 (H-3, dd, *J* = 4.3, 1.1 Hz), and a hydrogen-bonded phenolic hydroxy group at δ_H_ 11.68 (7-OH, s). The ^13^C-NMR and DEPT spectra of **1** revealed 14 carbon resonances attributable to two conjugated carbonyl groups [δ_C_ 186.5 (C-5) and 183.8 (C-10)], five sp^2^ quaternary carbons δ_C_ 162.2 (C-7), 155.2 (C-10a), 132.1 (C-9a), 118.8 (C-4a), 114.1 (C-5a), three sp^2^ methines δ_C_ 137.2 (C-6), 124.7 (C-8), 119.3 (C-9), three sp^3^ methines δ_C_ 76.2 (C-2), 66.9 (C-3), 65.1 (C-4), and one methyl δ_C_ 16.7 (C-11). As six of nine degrees of unsaturation account for four double bonds (eight sp^2^ carbons), and two carbonyl groups, the remaining degrees of unsaturation requires that **1** be a tricyclic system. The HMBC correlations (Figure 2) from H-6 to C-5, C-8, C-9a, and C-7, H-8 to C-7 and C-9a, H-9 to C-7 and C-10, and 7-OH to C-7 and C-8 suggested the presence of a 6-hydroxy-1,4-naphthoquinone moiety [16]. The location of the hydroxyl at C-7 was supported by HMBC correlations from 7-OH (δ_H_ 11.68) to C-5a, C-7 and C-8. Furthermore, the ^1^H-^1^H COSY spectrum (Figure 2) of **1** indicated the following fragments H_3_-11/H-2 and H-3/H-4, together with the HMBC correlations from H-3 to C-2, C-4a and C-11, and H-4 to C-4a, C-10a implied the remaining monocyclic was a 2*H*-pyran ring with a methyl at C-2 and OH at C-3 and C-4. The abovementioned information was similar to those of the co-isolated known compound (+)-cryptosporin (**3**), except for the OH at C-9 in **3** was locked at C-7 in **1**. Therefore, the planar structure of **1** was confirmed.

The relative configuration of **1** was established by the comparison of its ^1^H-NMR coupling constants [H-2 (*J* = 6.7, 1.0 Hz), H-3 (*J* = 4.3, 1.1 Hz), H-4 (*J* = 4.3, 0.9 Hz)] with those of **3** [H-2 (*J* = 6.5, 1.4 Hz), H-3 (*J* = 4.6, 2.1 Hz), H-4 (*J* = 4.6, 1.0 Hz)]. This indicated that these protons were cofacial and designated as β-oriented, whereas the Me-11 was α-oriented in **1**. The absolute configuration of **1** was determined by the CD spectrum. The absolute configuration of (+)-cryptosporin (**3**) was established by synthesis and its CD spectra data was reported in the literature [17]. The CD spectrum (Figure S19) of **1** showed a positive Cotton effect at 287, 314, 370 nm and a negative Cotton effect at 239, 425 nm, which was similar to that of **3**. Thus, the absolute stereochemistry of **1** was established as 2*R*,3*R*,4*R* and given the trivial name as acaromycin A.

Compound **2** was obtained as a light pink oil. The HRESIMS (*m*/*z* 278.0845, [M + H]^+^, calcd. for C_14_H_16_NO_3_S, 278.0851) allowed the molecular formula C_14_H_15_NO_3_S to be assigned to **2**, corresponding to eight degrees of unsaturation. The IR spectrum exhibited absorption bands for OH (3309 cm^−1^) and carboxyl groups (1732 and 1716 cm^−1^). The 1D NMR data of **2** (Table 1), in combination with the HSQC experiment revealed 14 carbon resonances, which showed representative signals for a 4-hydroxyphenylacetic acid moiety [δ_C_ 173.7, 157.6, 131.3, 126.1, 116.3, 41.2; δ_H_ 7.02 (2H, d, *J* = 8.6 Hz), 6.70 (2H, d, *J* = 8.6 Hz), 3.51 (s)]. This assumption was further confirmed by the HMBC correlations from H-2 to C-1, C-3 and C-8, H-5 to C-3 and C-7, and H-8 to C-6 and C-4. Besides, the ^1^H-NMR of **2** also exhibited a methyl signal at δ_H_ 2.24 (3H, s), two methylene protons at δ_H_ 3.02 (2H, t, *J* = 7.1 Hz) and 3.84 (2H, m), and an olefinic proton due to the thiazole ring at δ_H_ 8.61 (1H, s), suggesting the presence of a 4-methyl-5-thiazoleethanol substructure in **2**, which has been reported in the literature [18]. Furthermore, the key COSY correlations of H-7′/H-8′ and HMBC correlations from H-2′ to C-4′ and C-5′, as well as Me-6′ to C-4′, and C-5′ also supported this deduce (Figure 2). Finally, the two substructures was connected by the HMBC correlations between H-8 to C-1 and C-5′. Thus, compound **2** was determined as depicted and given the trivial name as acaromyester A.

The known compound (+)-cryptosporin (**3**) was identified by comparison of its NMR data with those in the literature [19].

### 2.2. In Vitro Growth Inhibition Assay

**Figure 3:**
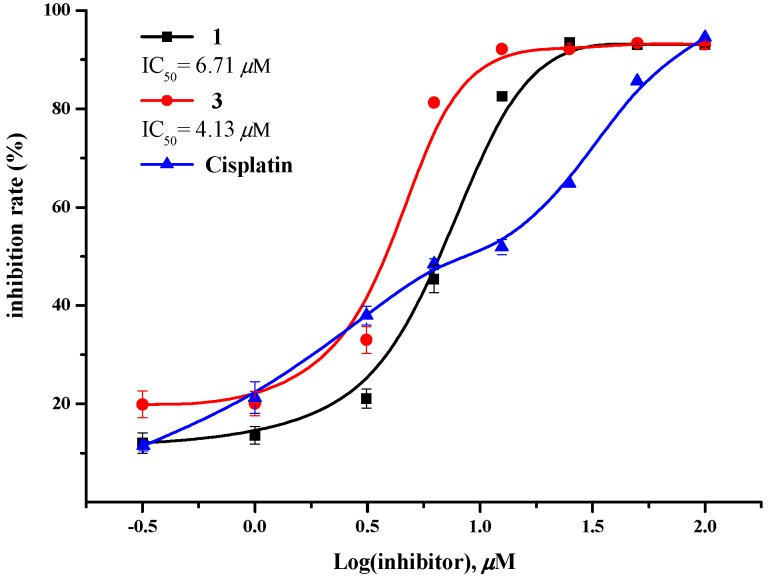
Growth inhibitory curves of compounds **1**, **3** and cisplatin (positive control) against MCF-7.

All of the isolates **1**–**3** were tested for *in vitro* growth inhibitory activities against the MCF-7, NCI-H460, SF-268 and HepG-2 tumor cell lines at an initial concentration of 100 µM. Then, compounds with inhibition greater than 50% were further analyzed to determine their corresponding IC_50_ values. Both **1** and **3** exhibited considerable inhibitory activities against the growth of all four cell lines (Table 2). Especially, the inhibitory effects of **1** and **3** against the MCF-7 cells were found to be comparable to that of cisplatin with IC_50_ values of 6.7 and 4.1 µM, respectively. The growth inhibitory curves of **1**, **3** and cisplatin against MCF-7 were presented in Figure 3.

## 3. Experimental Section

### 3.1. General Procedures

Optical rotations were measured on an MCP-500 spectropolarimeter (Anton Paar, Graz, Austria). Circular dichroism (CD) measurements were carried out under N_2_ gas on a Chirascan circular dichroism spectrometer (Applied Photophysics Ltd., Surrey, UK). IR spectra were recorded on a IR Affinity-1 spectrophotometer (Shimadzu, Kyoto, Japan). UV spectra were measured on a Shimadzu UV-2600 UV-visible spectrophotometer. NMR spectra were recorded on a Bruker Avance-500 spectrometer (Bruker Corporation, Fremont, CA, USA) with the signal of tetramethylsilane as an internal standard at 500/125 MHz, respectively. HRESIMS were measured on a Thermo MAT95XP high resolution mass spectrometer (Thermo Fisher Scientific, Bremen, Germany) and ESIMS on a 1290-6430A Triple Quad LC/MS (Agilent Technologies, (Agilent Technologies Inc., California, CA, USA). Column chromatography (CC) was performed on silica gel (200–300 mesh, Qingdao Haiyang Chemical Group Co., Qingdao, China), Lichroprep C_18_ reversed-phase (RP-18) silica gel (40–63 µm, Merck, Darmstadt, Germany) and Sephadex LH-20 (Pharmacia Fine Chemical Co. Ltd., Uppsala, Sweden). Thin-Layer Chromatography (TLC) was conducted with precoated glass plates GF-254 (Qingdao Haiyang Chemical Group Co.). TLC spots were visualized under UV light and by dipping into 10% H_2_SO_4_ in alcohol, followed by heating. Solvents were of the industrial purity and distilled prior to use.

### 3.2. Fungal Material

The marine fungal strain FS121 was isolated from a marine sediment sample, which was collected in the South China Sea (18°44.606′ N, 119°44.263′ E; 3415 m depth) in September 2011. The strain was identified by the sequence analysis of the rDNA ITS (internal transcribed spacer) region. The sequence of the ITS region of the strain FS121 has been submitted to GenBank (Accession No. KT989306). By using BLAST (nucleotide sequence comparison program) to search the GenBank database, FS121 has 99.8% similarity with *Acaromyces ingoldii* CBS 10536 (Accession No. AM991023). The strain is preserved at the Guangdong Provincial Key Laboratory of Microbiology Culture Collection and Application, Guangdong Institute of Microbiology.

### 3.3. Fermentation, Extraction and Compound Isolation

The fungal strain FS121 was maintained on potato dextrose agar (PDA) medium at 28 °C for 7 days, and then three pieces (0.5 × 0.5 cm^2^) of mycelial agar plugs were inoculated into 20 × 500 mL Erlenmeyer flasks, each containing 250 mL potato dextrose broth (potato 20%, glucose 2%, K_2_HPO_4_ 0.3%, MgSO_4_•7H_2_O 0.15%, vitamin B_1_ 10 mg/L, sea salt 1.5%). After 6 days of incubation at 28 °C on a rotary shaker at 120 r/m, 20 mL seed cultures were aseptically transferred into each of a total of 220 flasks (1000 mL) containing 500 mL of potato dextrose broth. The liquid cultivation that followed was kept for 7 days at 28 °C and 120 r/m on a rotary shaker. The culture (110 L) was centrifuged to give the broth and mycelia. The broth was exhaustively extracted with EtOAc four times, then the EtOAc layers were combined and evaporated under reduced pressure at a temperature not exceeding 40 °C to yield a dark brown gum (35.8 g). The crude extract was subjected to silica gel (200–300 mesh) column chromatography (CC) with a gradient system of increasing polarity (petroleum ether/EtOAc, 50:1→1:2) to afford 33 fractions, Fr.1–Fr.33. Fr.22 eluting with petroleum ether/EtOAc (1:2) was further fractionated by CC on Sephadex LH-20 (CH_2_Cl_2_/MeOH, 1:1) to yield three subfractions, Frs. 22-1, 22-2 and 22-3. Then, Fr. 22-1 was followed by CC over RP-18 silica gel (MeOH/H_2_O, 1:4) to yield Fr.22-1-3, then followed by preparative TLC on a silica gel plate (20 × 20 cm) developed with CH_2_Cl_2_/MeOH (10:1) to obtain compound **1** (2.6 mg). Fr.16 eluting with petroleum ether/EtOAc (2:1) was subjected to CC on Sephadex LH-20 (CH_2_Cl_2_/MeOH, 1:1) to afford compound **2** (22 mg). Fr.20 was subjected to CC on Sephadex LH-20 (CH_2_Cl_2_/MeOH, 1:1) to give three subfractions: Fr.20-1, Fr.20-2 and Fr.20-3. Fr.20-1 was purified by CC over RP-18 silica gel (MeOH/H_2_O, 1:1) to yield compound **3** (3.8 mg).

### 3.4. Compound Characterization

*Acaromycin A* (**1**): orange amorphous powder; ${\left[\alpha \right]}_{D}^{25}$ +102.0 (*c* 0.1, MeOH); UV (MeOH) *λ*_max_ (log ε) 194 (3.69), 204 (4.05), 286 (3.67) nm; CD (*c* 1.8 × 10^−3^, MeOH) λ(Δε) 474 (0), 425 (−0.25), 405 (0), 370 (0.76), 314 (0.19), 287 (2.1), 257 (0), 239 (−0.67), 221 (0) nm; IR ν_max_ 3498, 1647, 1625, 1257, 1026, 792 cm^−1^; ^1^H- and ^13^C-NMR, see Table 1; HRESIMS *m*/*z* [M + Na]^+^ 299.0533 (calcd for C_14_H_12_O_6_Na, 299.0532).

*Acaromyester A* (**2**): light pink oil; UV (MeOH) λ_max_ (log ε) 202 (4.24), 225 (4.08), 277 (3.44) nm; IR ν_max_ 3309, 3178, 1732, 1716, 1516, 1236, 1145, 804, 783 cm^−1^; ^1^H- and ^13^C-NMR, see Table 1; HRESIMS *m*/*z* [M + H]^+^ 278.0845 (calcd for C_14_H_16_NO_3_S, 278.0851).

### 3.5. In Vitro Growth Inhibition Assay

Cell growth inhibitory effects of compounds **1**–**3** were tested against four tumor cell lines, including MCF-7 (human breast adenocarcinoma cell line), NCI-H460 (human non-small cell lung cancer cell line) and SF-268 (human glioma cell line), HepG-2 (human hepatomacarcinoma cell line) by the SRB method [20]. Cells (180 μL) with a density of 3 × 10^4^ cells/mL of media were seeded onto 96-well plates and incubated for 24 h at 37 °C, 5% CO_2_. Various concentrations of compounds (20 μL) were added to the plate wells, and plates were further incubated for 72 h. After incubation, cell monolayers were fixed with 50% (*w*/*v*) trichloroacetic acid (50 μL) and stained for 30 min with 0.4% (*w*/*v*) SRB dissolved in 1% acetic acid. Unbound dye was removed by washing repeatedly with 1% acetic acid. The protein-bound dye was dissolved in 10 mM Tris base solution (200 µL) for OD determination at 570 nm using a microplate reader. Cisplatin was used as a positive control. All data were obtained in triplicate and are presented as means ± S.D. IC_50_ values were calculated with the SigmaPlot 10.0 software (San Jose, California, CA, USA) using a nonlinear curve-fitting method.

## 4. Conclusions

In summary, we have described herein the isolation and structural elucidation of a new 3,4-dihydro-2*H*-naphtho-[2,3-*b*]pyran-5,10-quinone and a new heterodimeric compound combining a hydroxyphenylacetate and a 4-methyl-5-thiazoleethanol substructure, which were isolated from the fermentation broth of *Acaromyces ingoldii* FS121. It merits attention that compounds **1** and **3** contained a 3,4-dihydro-2*H*-naphtho-[2,3-*b*]pyran-5,10-quinone core framework, which exhibited a wide range of biological activities [21]. The cell growth inhibitory effects of compounds **1**–**3** were evaluated, wherein compound **1** and **3** showed considerable activities against MCF-7, NCI-H460, SF-268 and HepG-2 tumor cell lines.

## Figures and Tables

**Table 1:** ^1^H- (500 MHz) and ^13^C- (125 MHz) NMR data of **1** in CDCl_3_ and **2** in CD_3_OD.

Position	1		Position	2	
	δ_C_	δ_H_ (*J* in Hz)		δ_C_	δ_H_ (*J* in Hz)
2	76.1, CH	4.30, q (6.7, 1.0)	1	173.7, C	
3	66.9, CH	4.03, dd (4.3, 1.1)	2	41.2, CH_2_	3.51, s
4	65.1, CH	4.96, d (4.3, 0.9)	3	126.1, C	
4a	118.8, C		4	131.3, CH	7.02, d (8.6)
5	186.5, C		5	116.3, CH	6.70, d (8.6)
5a	114.1, C		6	157.6, C	
6	137.2, CH	7.60, s	7	116.3, CH	6.70, d (8.6)
7	162.2, C		8	131.3, CH	7.02, d (8.6)
8	124.7, CH	7.23, dd (5.6, 4.0)	2′	152.4, CH	8.75, s
9	119.3, CH	7.61, d (1.9)	4′	150.6, C	
9a	132.1, C		5′	129.1, C	
10	183.9, C		6′	14.5, CH_3_	2.33, s
10a	155.2, C		7′	26.5, CH_2_	3.12, t (6.3)
11	16.7, CH_3_	1.65, d (6.7)	8′	65.5, CH_2_	4.26, t (6.3)
3-OH		2.92, brs			
4-OH		5.02, s			
7-OH		11.68, s			

**Table 2:** Growth-inhibition effects of compounds **1** and **3**.

Compounds	IC_50_ (µM) ($\bar{x}\pm s,n=3$)			
	MCF-7	NCI-H460	SF-268	HepG-2
**1**	6.7 ± 1.6	10.0 ± 0.1	7.8 ± 0.5	7.3 ± 1.8
**3**	4.1 ± 0.3	6.0 ± 0.1	5.7 ± 0.1	5.7 ± 0.6
Cisplatin *	5.8 ± 0.4	1.3 ± 0.1	1.9 ± 0.1	1.7 ± 0.1

* Positive control.

## Supplementary Materials

Supplementary materials can be accessed at: http://www.mdpi.com/1420-3049/21/4/371/s1.

## References

[1] Bhadury P., Mohammad B.T., Wright P.C. (2006). The current status of natural products from marine fungi and their potential as anti-infective agents. J. Ind. Microbiol. Biot..

[2] Blunt J.W., Copp B.R., Munro M.H.G., Northcote P.T., Prinsep M.R. (2003). Marine natural products. Nat. Prod. Rep..

[3] Blunt J.W., Copp B.R., Munro M.H.G., Northcote P.T., Prinsep M.R. (2004). Marine natural products. Nat. Prod. Rep..

[4] Debbab A., Aly A.H., Lin W.H., Proksch P. (2010). Bioactive compounds from marine bacteria and fungi. Microb. Biotechnol..

[5] Faulkner D.J. (2002). Marine natural products. Nat. Prod. Rep..

[6] Jiang W., Ye P., Che C.-T.A., Wang K., Liu P., He S., Wu X., Gan L., Ye Y., Wu B. (2013). Two novel hepatocellular carcinoma cycle inhibitory cyclodepsipeptides from a hydrothermal vent crab-associated fungus *Aspergillus clavatus* C2WU. Mar. Drugs.

[7] Saleem M., Ali M.S., Hussain S., Jabbar A., Ashraf M., Lee Y.S. (2007). Marine natural products of fungal origin. Nat. Prod. Rep..

[8] Sun L., Li D.L., Tao M.H., Chen Y.C., Dan F.J., Zhang W.M. (2012). Scopararanes C-G: new oxygenated pimarane diterpenes from the marine sediment-derived fungus *Eutypella scoparia* FS26. Mar. Drugs.

[9] Wu B., Wu X., Sun M., Li M. (2013). Two novel tyrosinase inhibitory sesquiterpenes induced by CuCl2 from a marine-derived fungus *Pestalotiopsis* sp. Z233. Mar. Drugs.

[10] Yamazaki H., Rotinsulu H., Kaneko T., Murakami K., Fujiwara H., Ukai K., Namikoshi M. (2012). A new dibenz[*b*,*e*]oxepine derivative, 1-hydroxy-10-methoxy-dibenz[*b*,*e*]oxepin-6,11-dione, from a marine-derived fungus, *Beauveria bassiana* TPU942. Mar. Drugs.

[11] Evidente A., Kornienko A., Cimmino A., Andolfi A., Lefranc F., Mathieu V., Kiss R. (2014). Fungal metabolites with anticancer activtity. Nat. Prod. Rep..

[12] Gomes N.G.M., Lefranc F., Kijjoa A., Kiss R. (2015). Can some marine-derived fungal metabolites become actual anticancer agents. Mar. Drugs.

[13] Kornienko A., Evidente A., Vurro M., Mathieu V., Cinmmino A., Evidente M., Otterlo W.A.L., Dasari R., Lefranc F., Kiss R. (2015). Toward a cancer drug of fungal origin. Med. Res. Rev..

[14] Mander L.N., Liu H.W. (2010). In natural product diversity from marine fungi. Comprehensive Natural Products II.

[15] Yang X.L., Chen Y.C., Li H.H., Zhang W.M. (2014). Molecular identification of 23 marine fungal strains and their activties against plant pathogenic fungi and cytotoxic activities. Biotechnol. Bull..

[16] Huang L., Zhao J., Guo S., Zhang C., Ma J. (2013). Bodipy derivatives as organic triplet photosensitizers for aerobic photoorganocatalytic oxidative coupling of amines and photooxidation of dihydroxylnaphthalenes. J. Org. Chem..

[17] Brade W., Vasella A. (1989). Synthesis of naphtho[2,3-*b*]pyrandiones: (‒)-cryptosporin. Helv. Chim. Acta.

[18] Park J.D., Kim M.W., Yoo S.J., Wee J.J. (1988). A thiazole and two β-carboline constituents from *Panax ginseng*. Arch. Pharm. Res..

[19] Closse A., Sigg H.P. (1973). Isolation and structure elucidation of cryptosporin. Helv. Chim. Acta.

[20] Skehan P., Storeng R., Scudiero D., Monks A., McMahon J., Vistica D., Warren J.T., Bokesch H., Kenney S., Boyd M.R. (1990). New colorimetric cytotoxicity assay for anticancer-drug screening. J. Natl. Cancer. Inst..

[21] Gupta R.B., Franck R.W. (1989). The total synthesis of (‒)-cryptosporin. J. Am. Chem. Soc..

