# Supplementary material for: Secondary Metabolites from the Deep-Sea Derived Fungus Acaromyces ingoldii FS121
Source: Molecules. 2016 Mar 29;21(4):371. doi: 10.3390/molecules21040371 (PMC6274379; doi:10.3390/molecules21040371)
Supplement: Supplementary file 1 [file molecules-21-00371-s001.pdf]

# Supplementary Materials: Secondary Metabolites from the Deep-Sea Derived Fungus *Acaromyces ingoldii* FS121

**Xiao-Wei Gao, Hong-Xin Liu, Zhang-Hua Sun, Yu-Chan Chen, Yu-Zhi Tan and Wei-Min Zhang**

**Contents**


**Figure S1.** $^1$H-NMR spectrum of (+)-acaromycin A (**1**) in $CDCl_3$.

**Figure S2.** $^{13}$C-NMR spectrum of (+)-acaromycin A (**1**) in $CDCl_3$.

**Figure S3.** DEPT 135 spectrum of (+)-acaromycin A (**1**) in $CDCl_3$.

**Figure S4.** HSQC spectrum of (+)-acaromycin A (**1**) in $CDCl_3$.

**Figure S5.** HMBC spectrum of (+)-acaromycin A (**1**) in $CDCl_3$.

**Figure S6.** $^1$H-$^1$H COSY spectrum of (+)-acaromycin A (**1**) in $CDCl_3$.

**Figure S7.** NOESY spectrum of (+)-acaromycin A (**1**) in $CDCl_3$.

**Figure S8.** HRESIMS spectrum of (+)-acaromycin A (**1**).

**Figure S9.** UV spectrum of (+)-acaromycin A (**1**).

**Figure S10.** IR spectrum of (+)-acaromycin A (**1**).

**Figure S11.** CD spectrum of (+)-acaromycin A (**1**)..

**Figure S12.** $^1$H-NMR spectrum of acaromyester A (**2**) in $CD_3OD$.

**Figure S13.** $^{13}$C-NMR spectrum of acaromyester A (**2**) in $CD_3OD$.

**Figure S14.** HSQC spectrum of acaromyester A (**2**) in $CD_3OD$.

**Figure S15.** HMBC spectrum of acaromyester A (**2**) in $CD_3OD$.

**Figure S16.** $^1$H-$^1$H COSY spectrum of acaromyester A (**2**) in $CD_3OD$.

**Figure S17.** HRESIMS spectrum of acaromyester A (**2**).

**Figure S18.** UV spectrum of acaromyester A (**2**).

**Figure S19.** IR spectrum of acaromyester A (**2**).

**Figure S20.** $^1$H-NMR spectrum of (+)-cryptosporin (**3**) in DMSO-$d_6$.

**Figure S21.** $^{13}$C-NMR spectrum of (+)-cryptosporin (**3**) in DMSO-$d_6$.

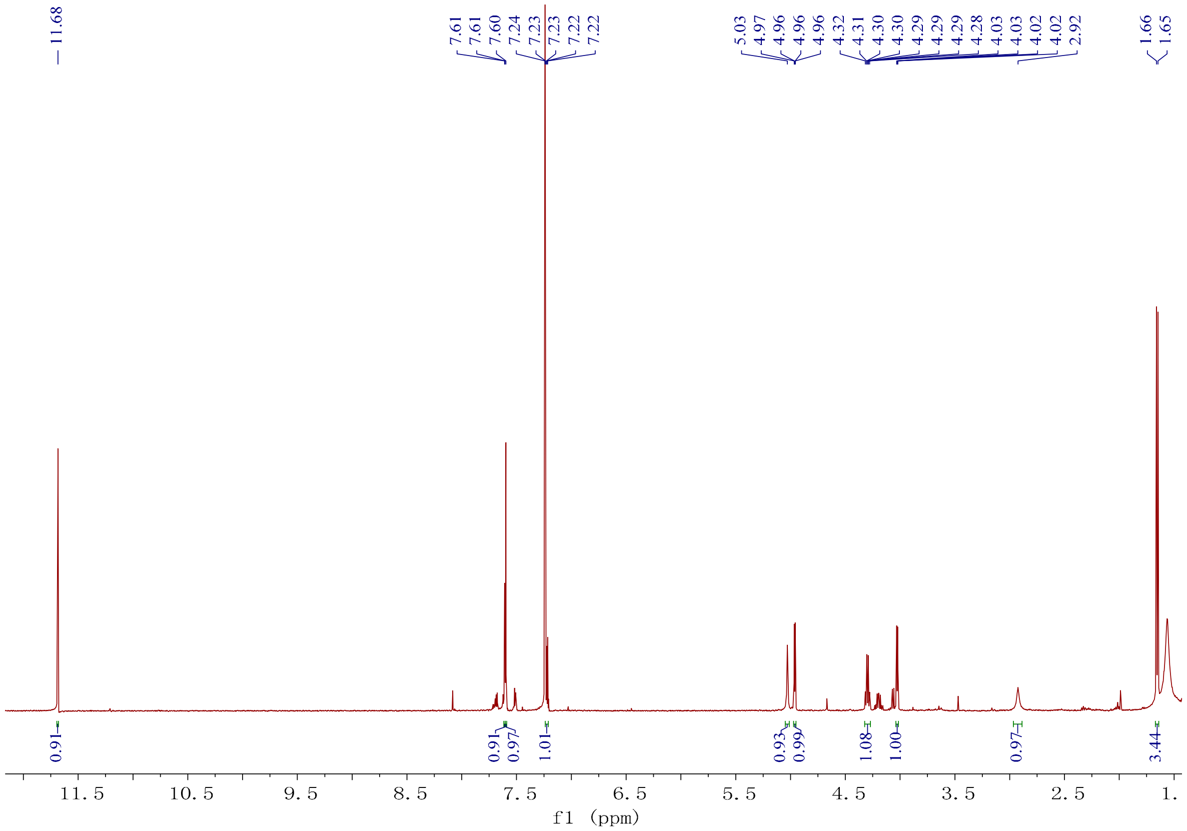


**Figure S1.** $^{1}$H-NMR spectrum of (+)-acaromycin A (**1**) in $CDCl_3$.

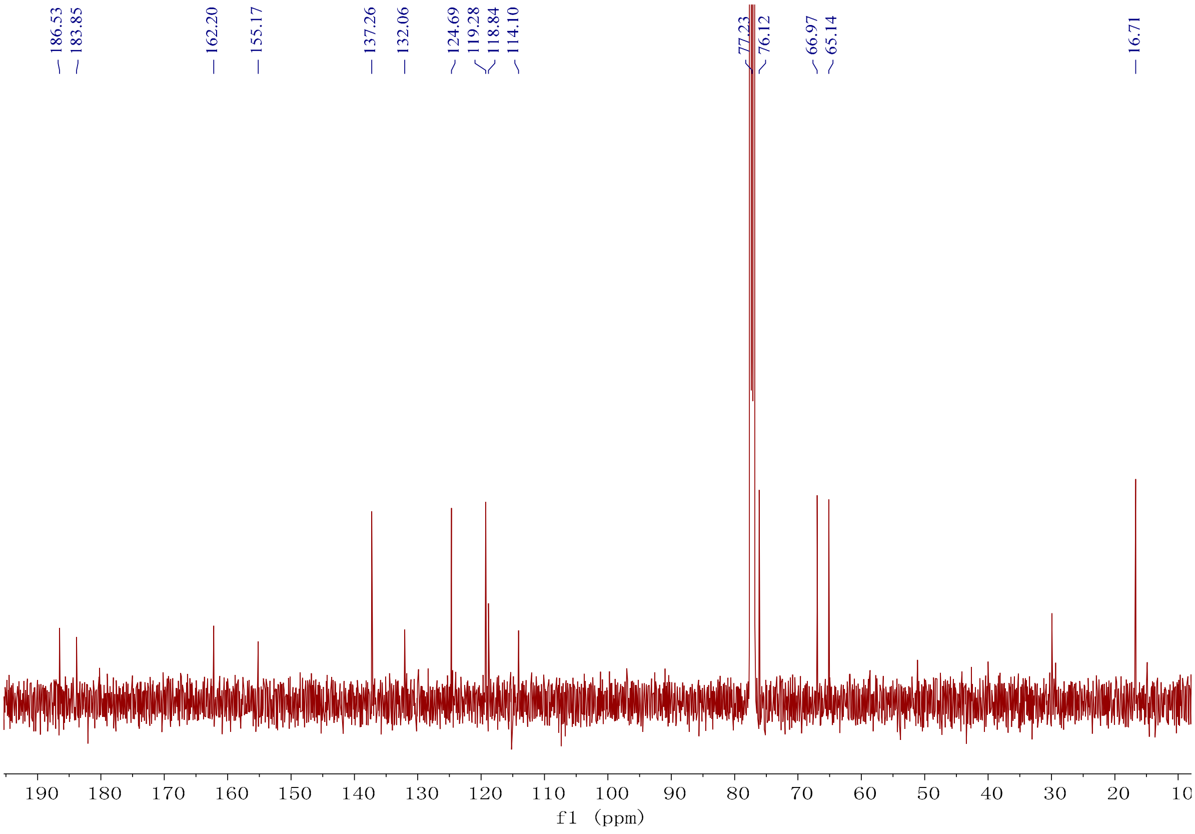


**Figure S2.** $^{13}$C-NMR spectrum of (+)-acaromycin A (**1**) in $CDCl_3$.

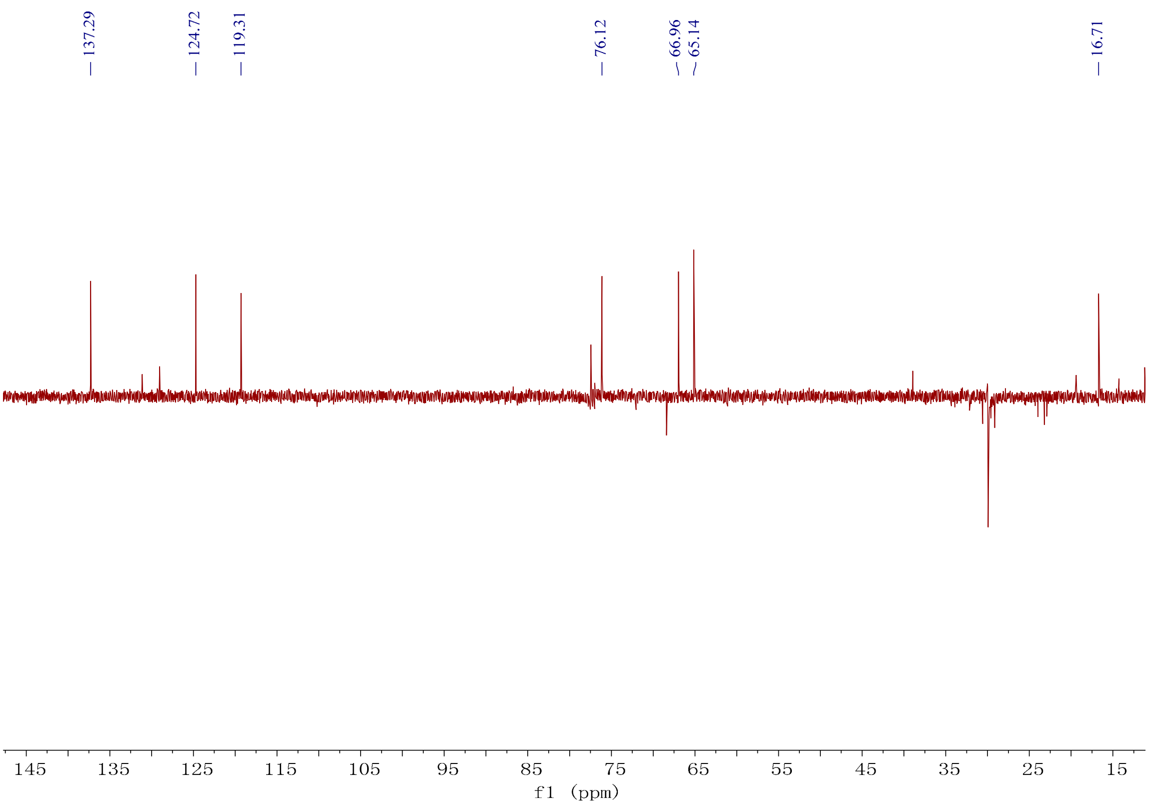


**Figure S3.** DEPT 135 spectrum of (+)-acaromycin A (**1**) in $CDCl_3$.

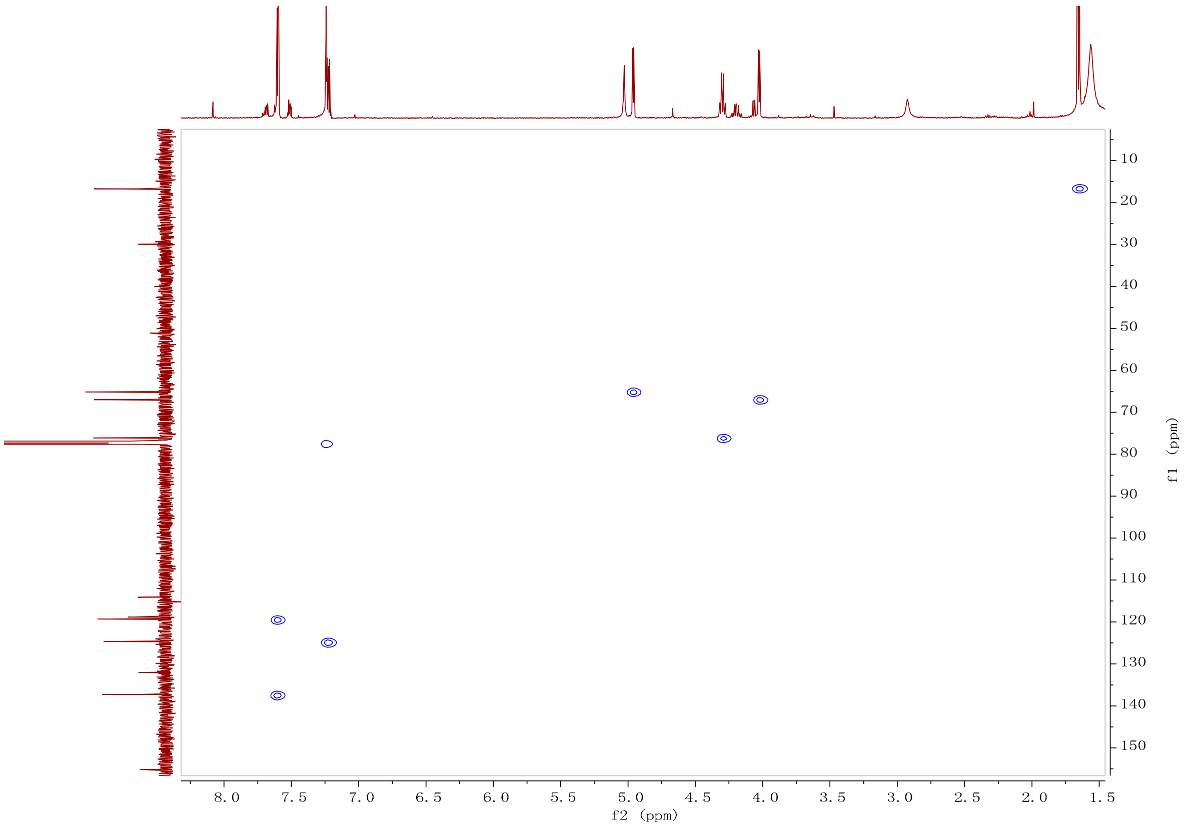


**Figure S4.** HSQC spectrum of (+)-acaromycin A (**1**) in $CDCl_3$.

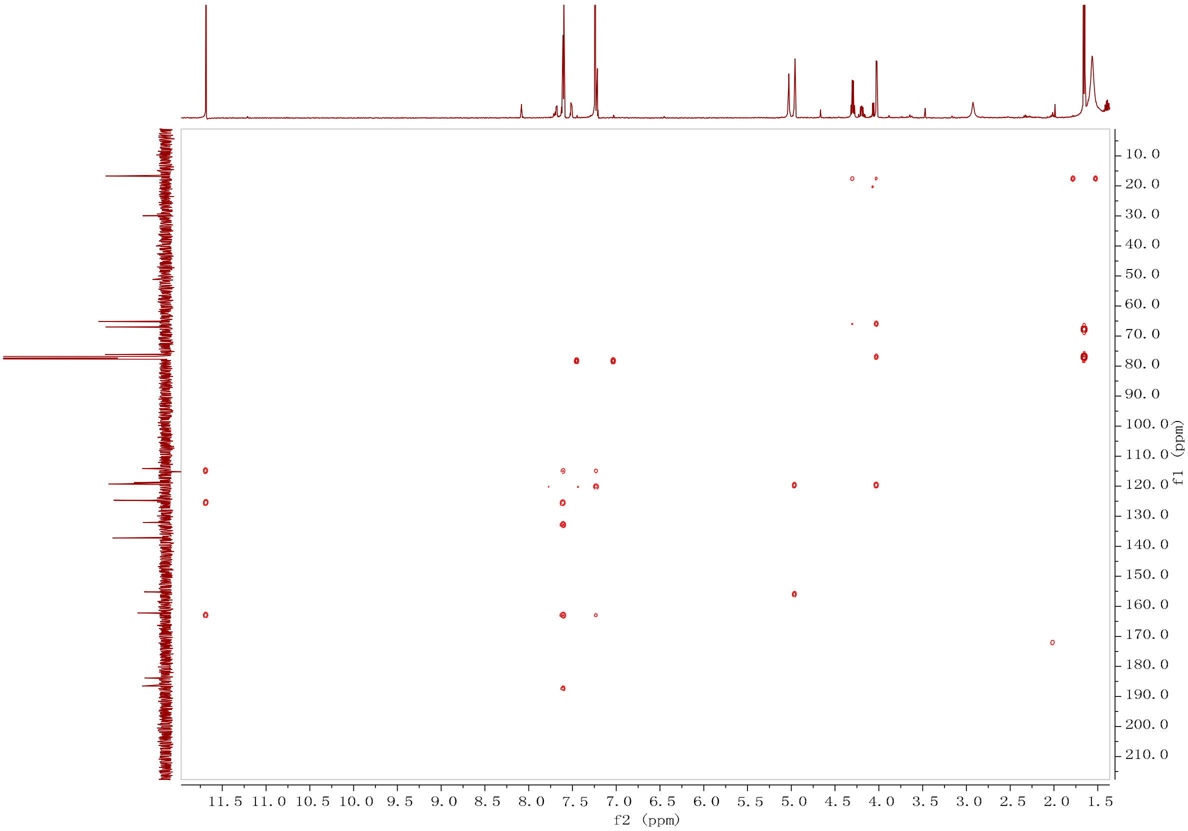


**Figure S5.** HMBC spectrum of (+)-acaromycin A (**1**) in $CDCl_3$.

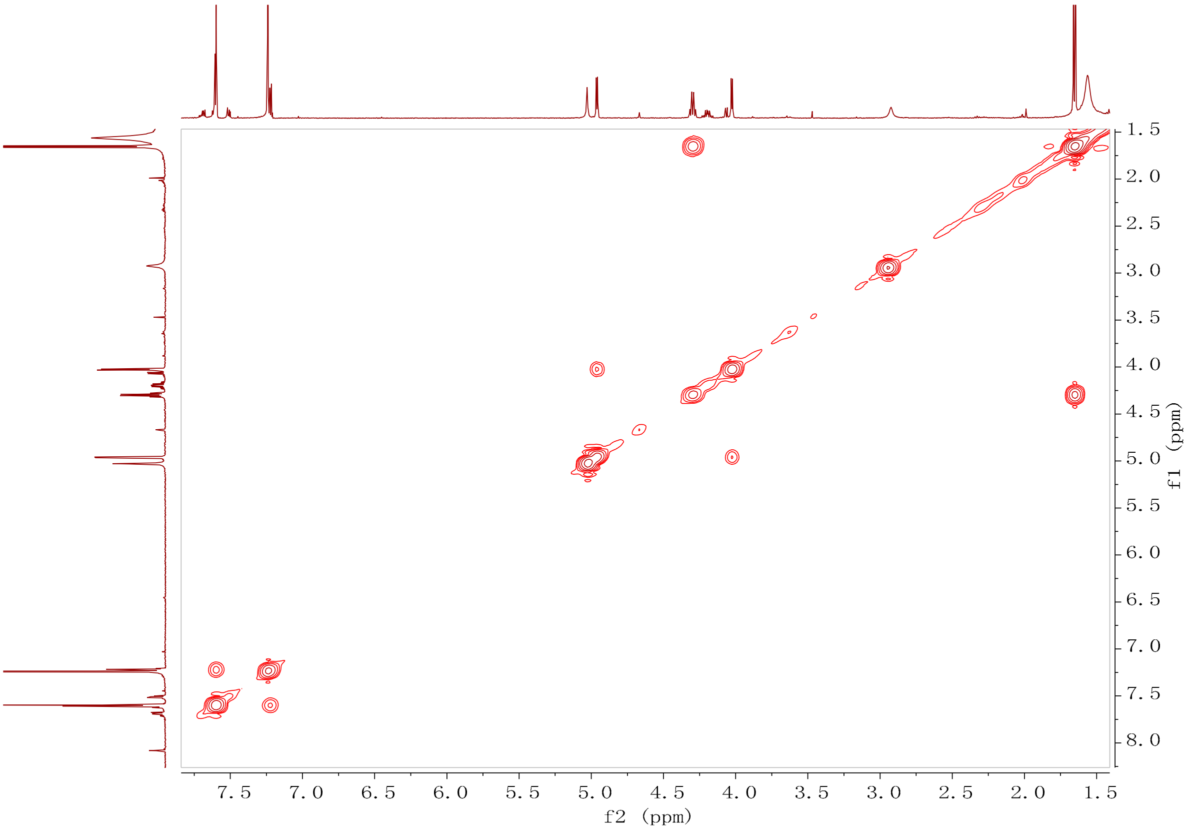


**Figure S6.** $^{1}$H-$^{1}$H COSY spectrum of (+)-acaromycin A (**1**) in $CDCl_3$.

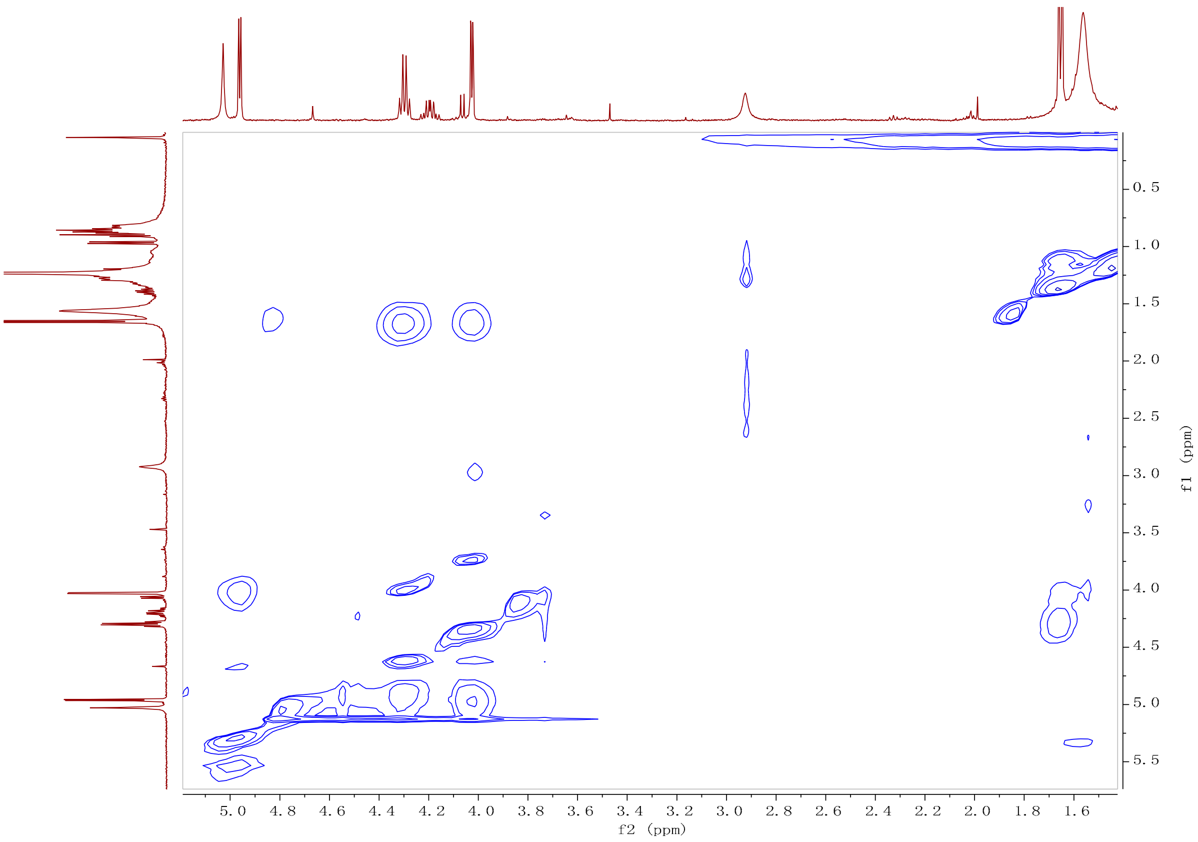


**Figure S7.** NOESY spectrum of (+)-acaromycin A (**1**) in $CDCl_3$.

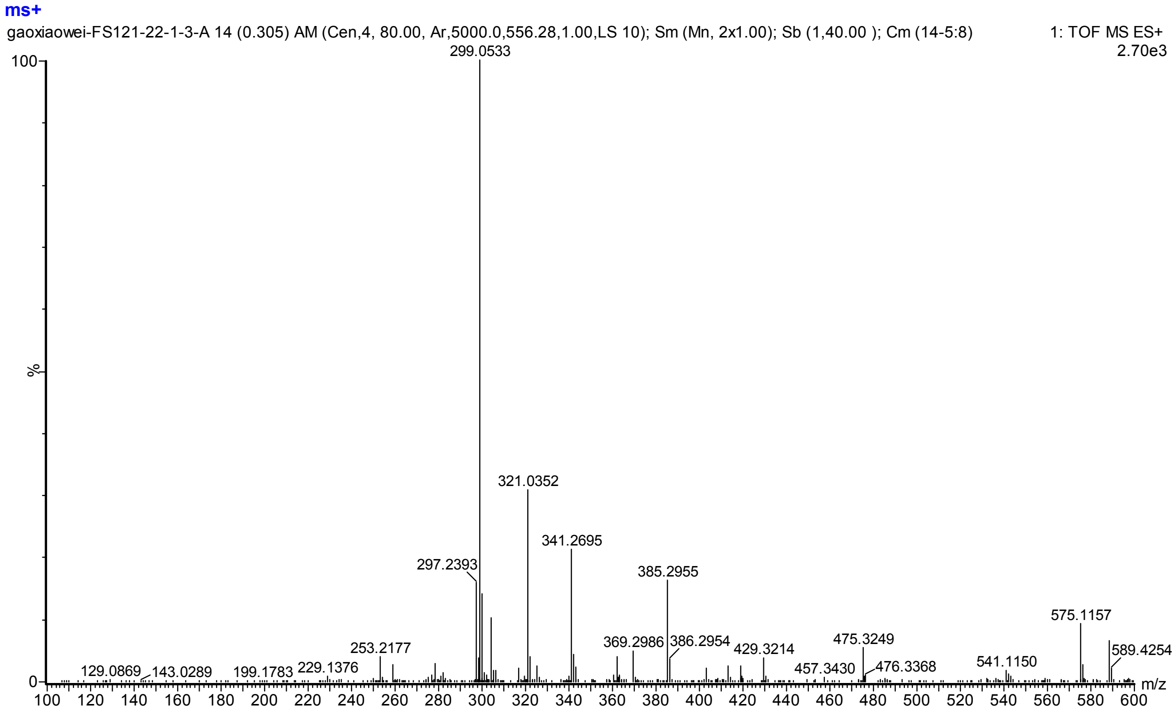


**Figure S8.** HRESIMS spectrum of (+)-acaromycin A (**1**).

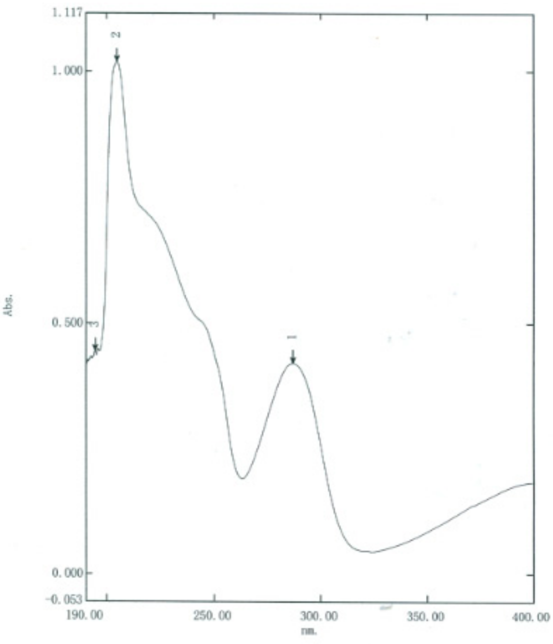


**Figure S9.** UV spectrum of (+)-acaromycin A (**1**).

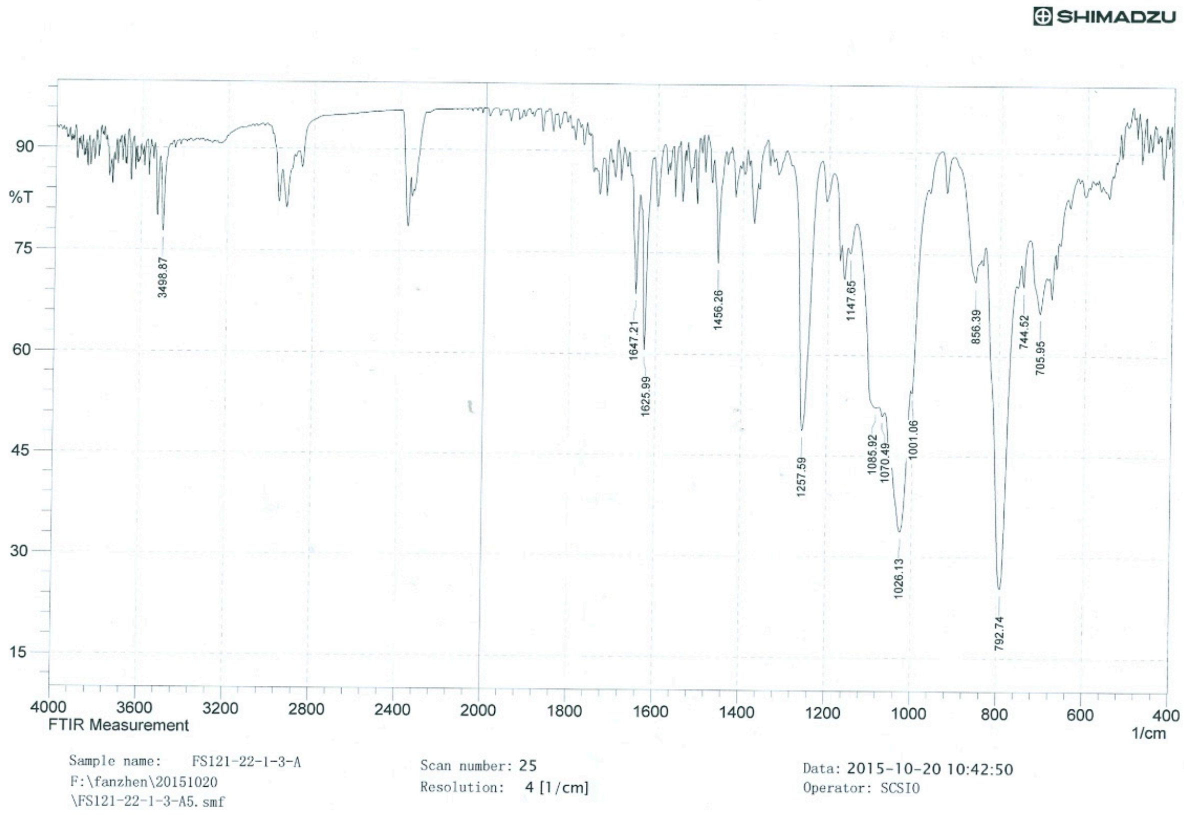


**Figure S10.** IR spectrum of (+)-acaromycin A (**1**).

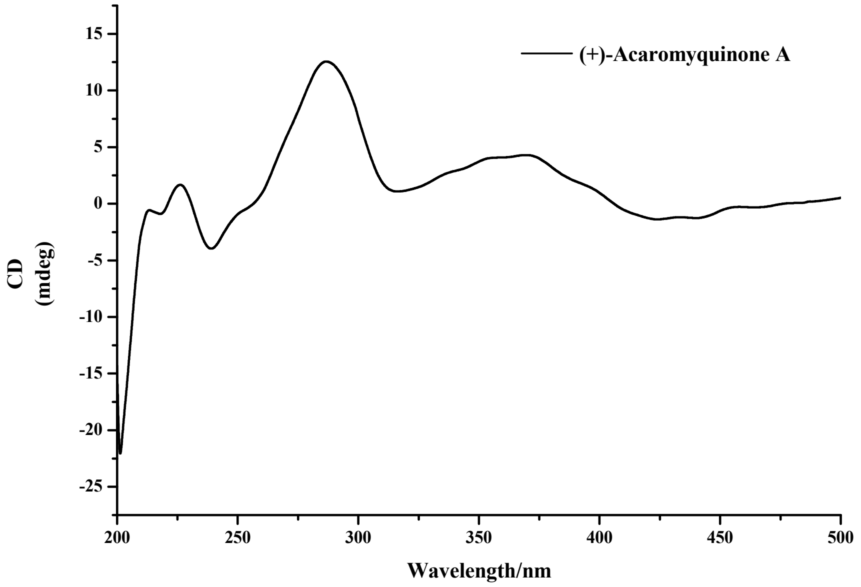


**Figure S11.** CD spectrum of (+)-acaromycin A (**1**).

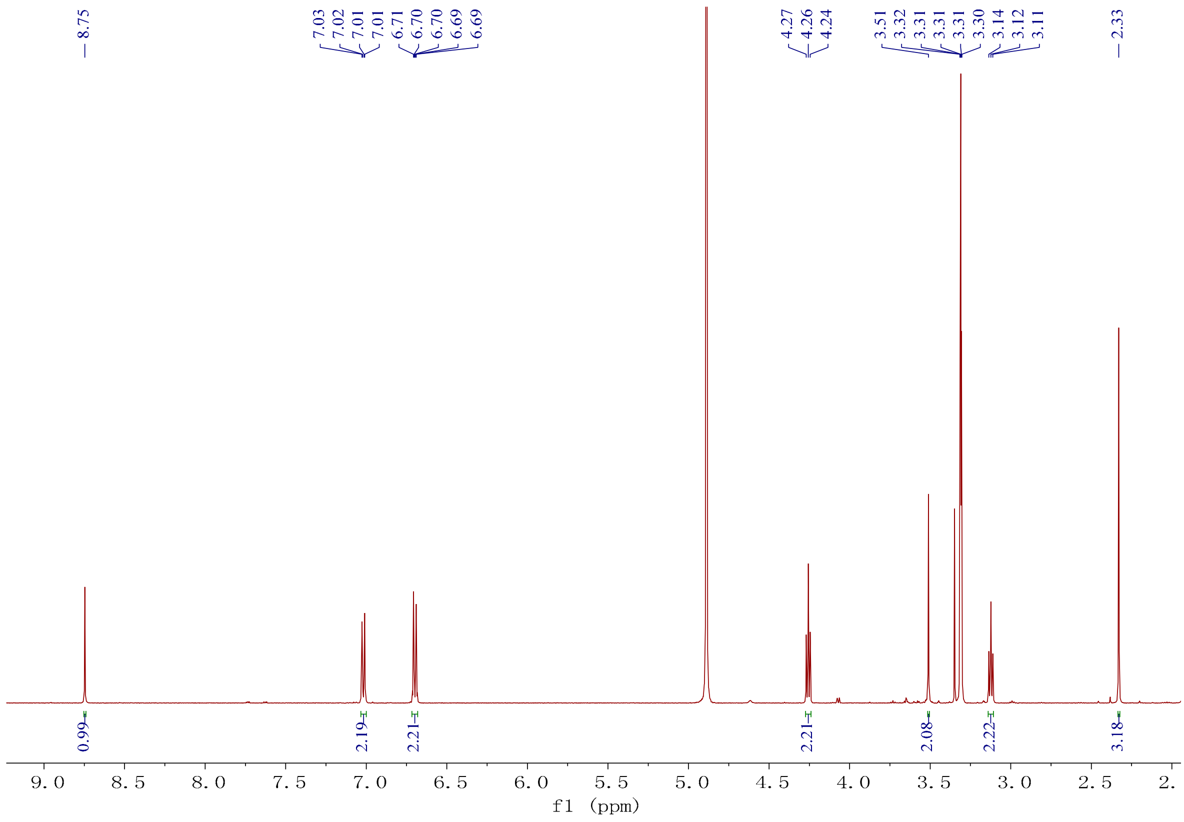


**Figure S12.** $^{1}H$-NMR spectrum of acaromyester A (**2**) in $CD_3OD$.

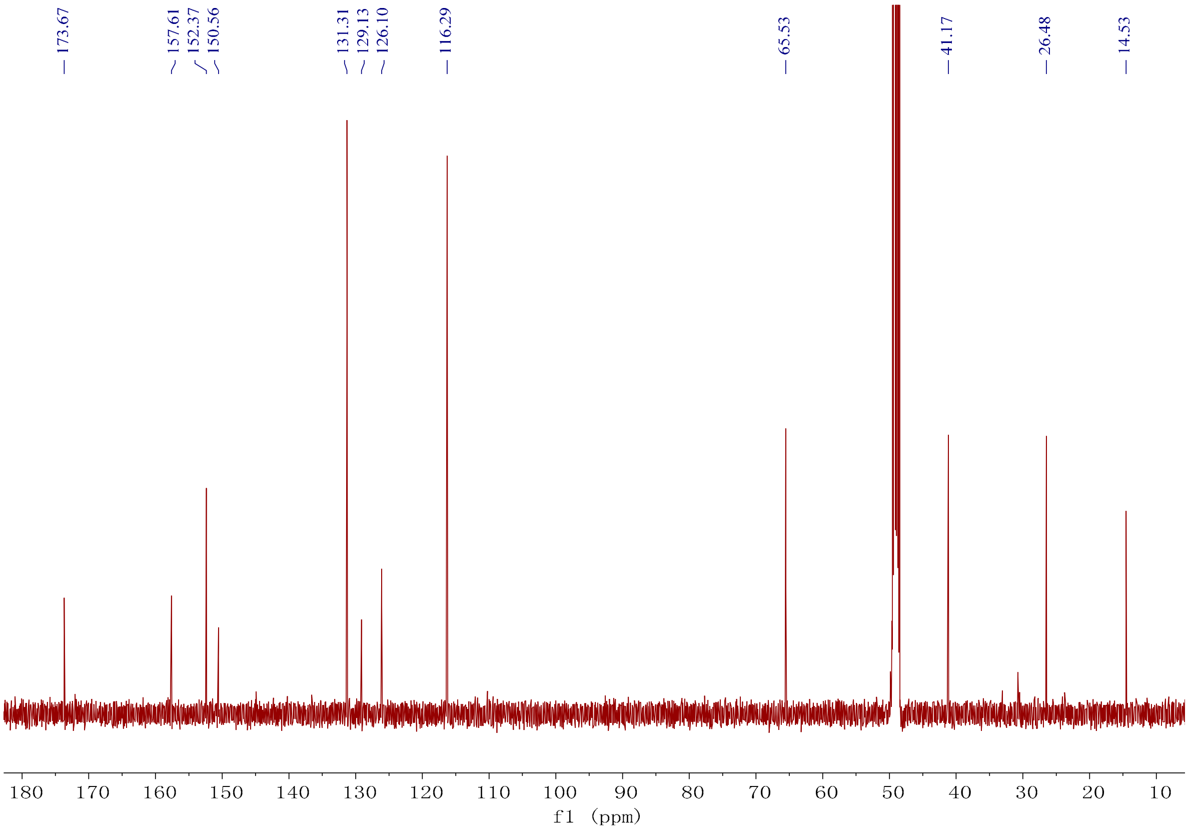


**Figure S13.** $^{13}$C-NMR spectrum of acaromyester A (**2**) in $CD_3OD$.

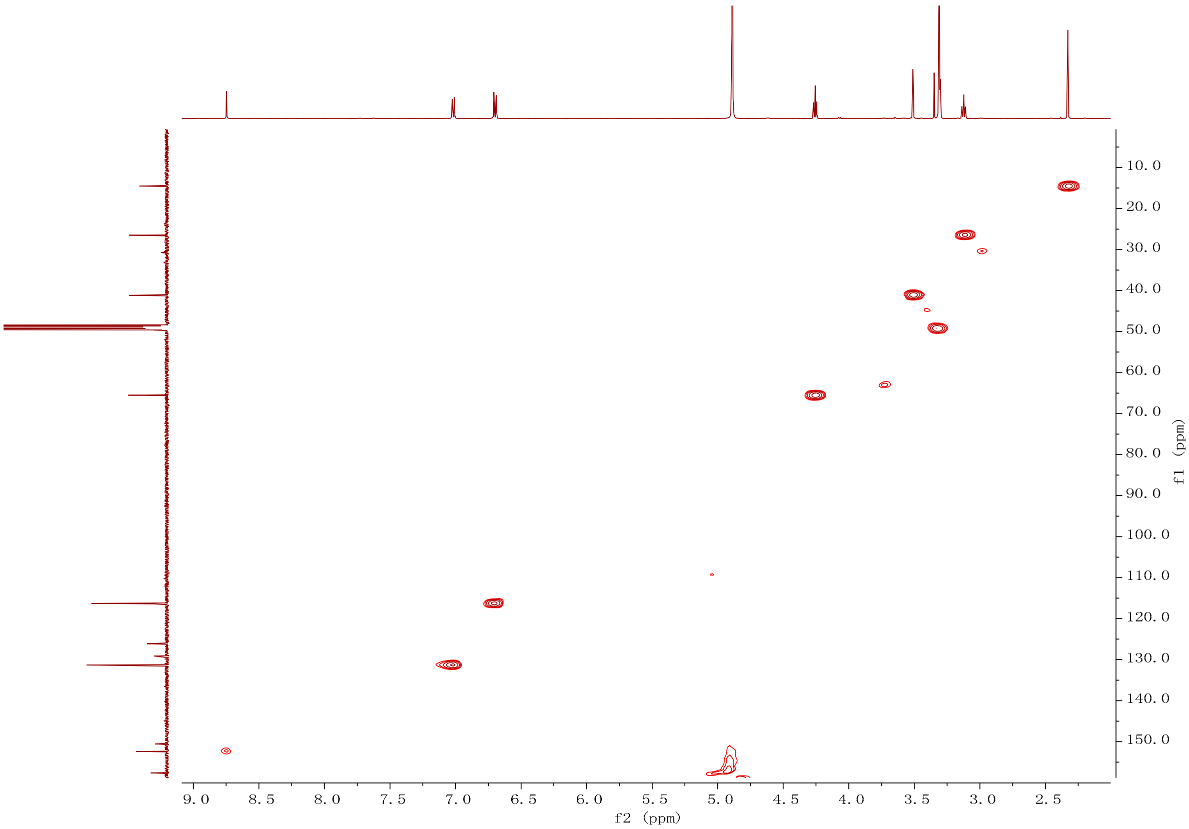


**Figure S14.** HSQC spectrum of acaromyester A (**2**) in $CD_3OD$.

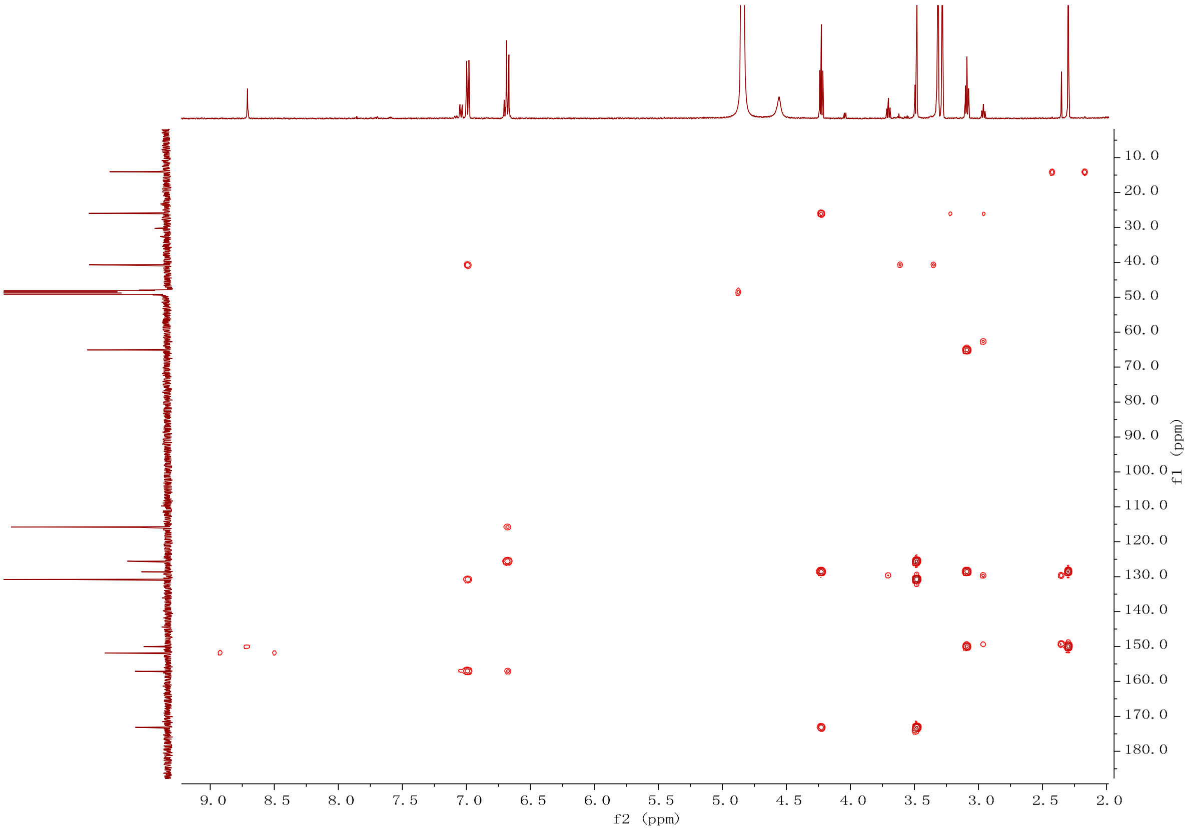


**Figure S15.** HMBC spectrum of acaromyester A (**2**) in $CD_3OD$.

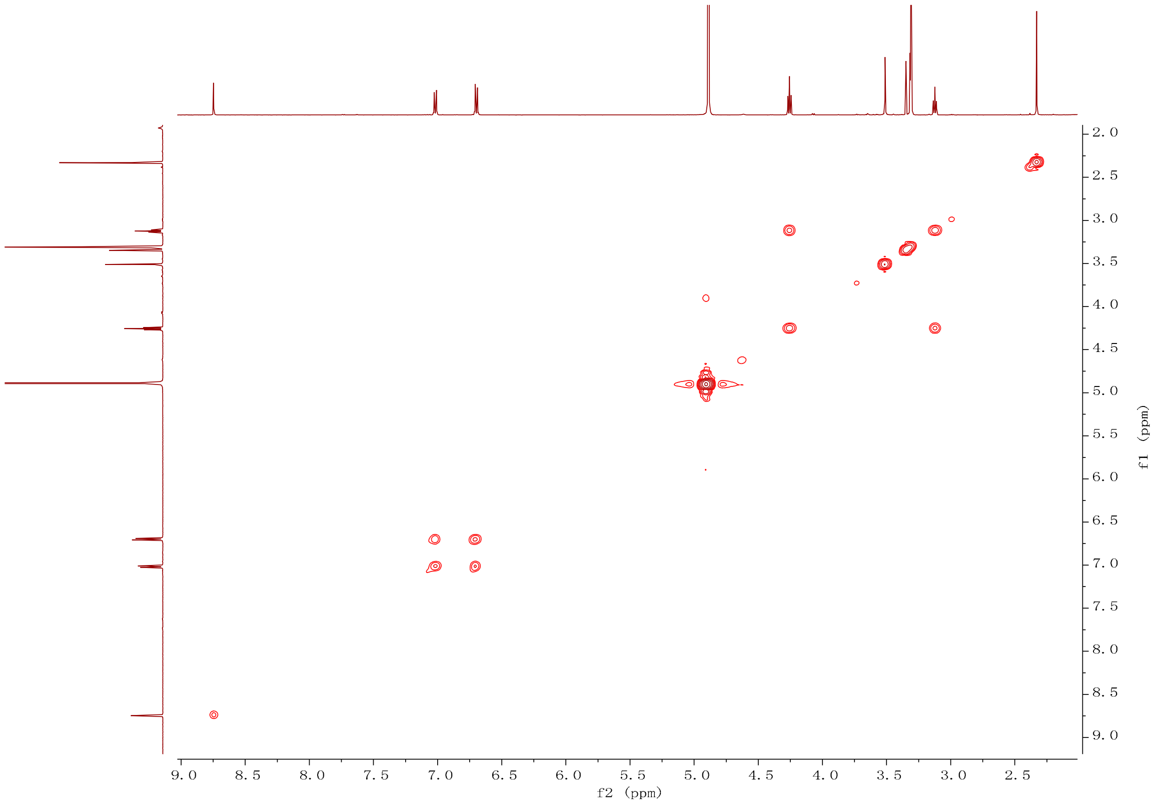


**Figure S16.** $^1H$-$^1H$ COSY spectrum of acaromyester A (**2**) in $CD_3OD$.

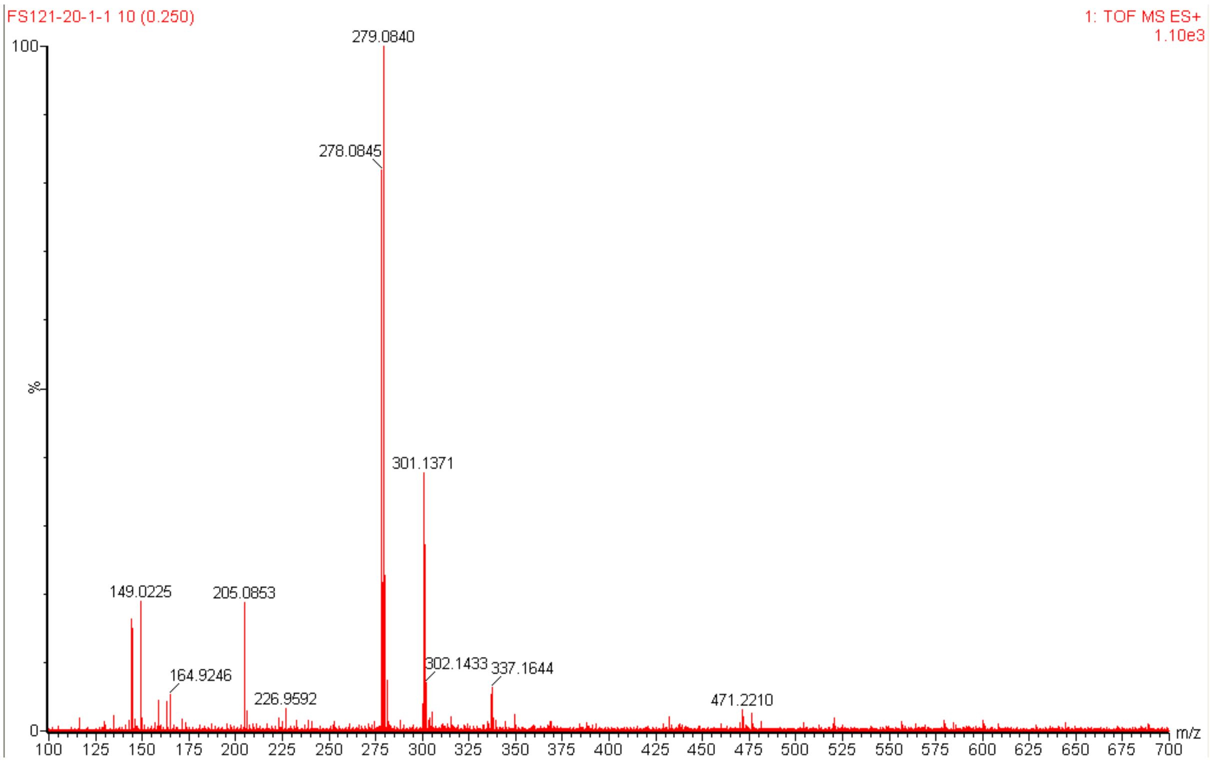


**Figure S17.** HRESIMS spectrum of acaromyester A (**2**).

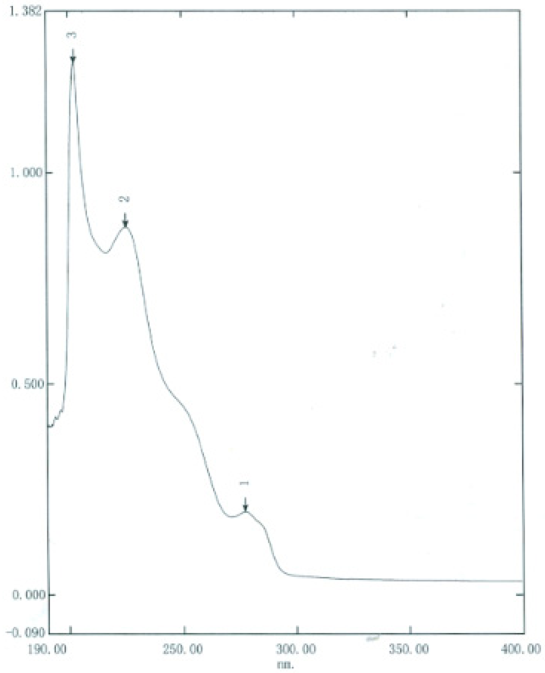


**Figure S18.** UV spectrum of acaromyester A (**2**).

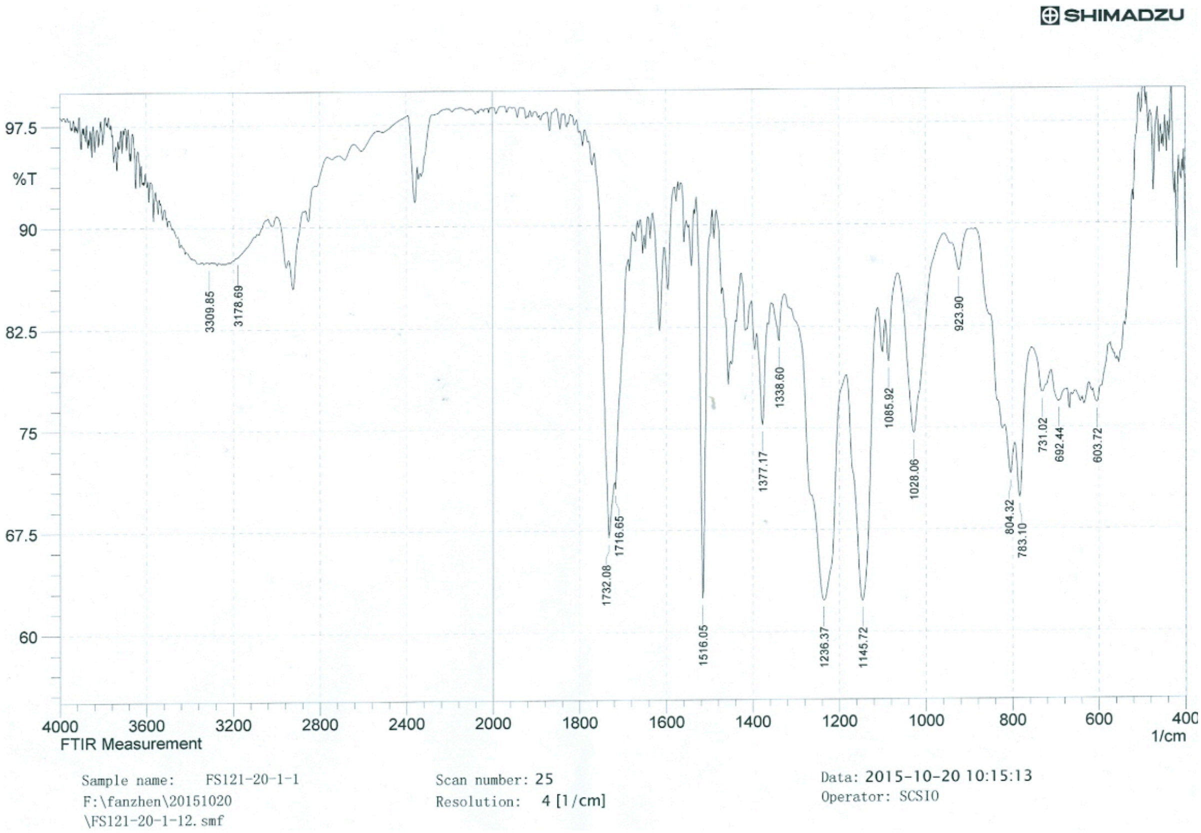


**Figure S19.** IR spectrum of acaromyester A (**2**).

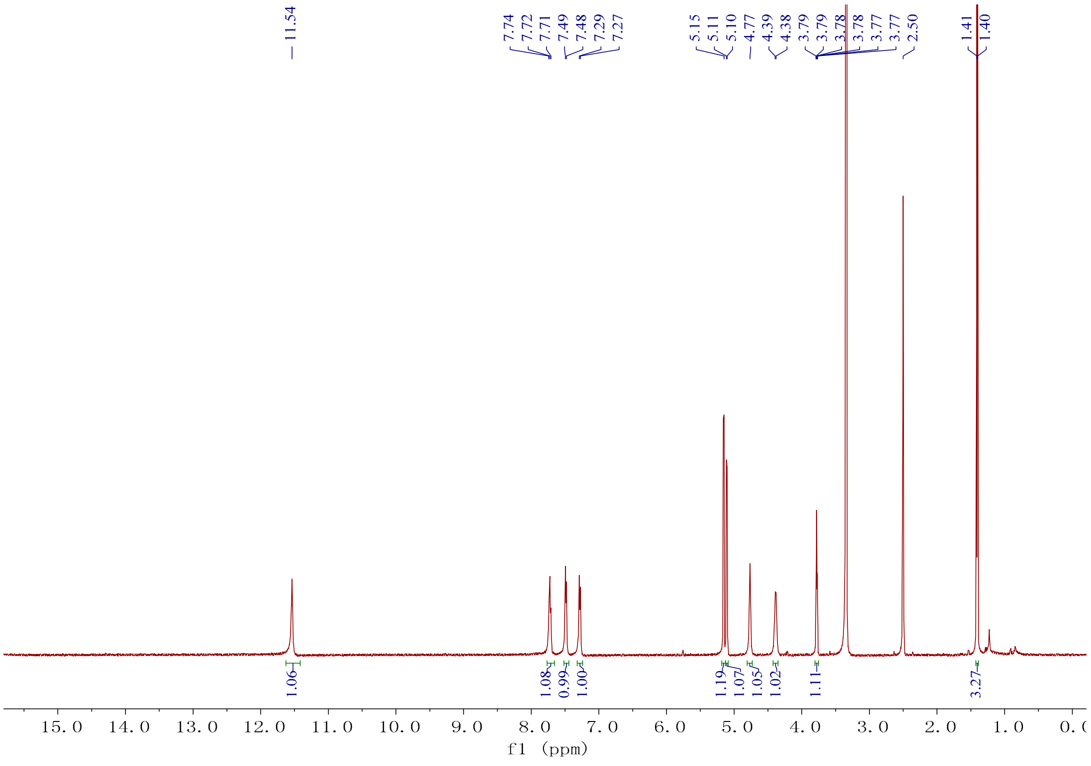


**Figure S20.** $^{1}$H-NMR spectrum of (+)-cryptosporin (**3**) in DMSO-$d_6$.

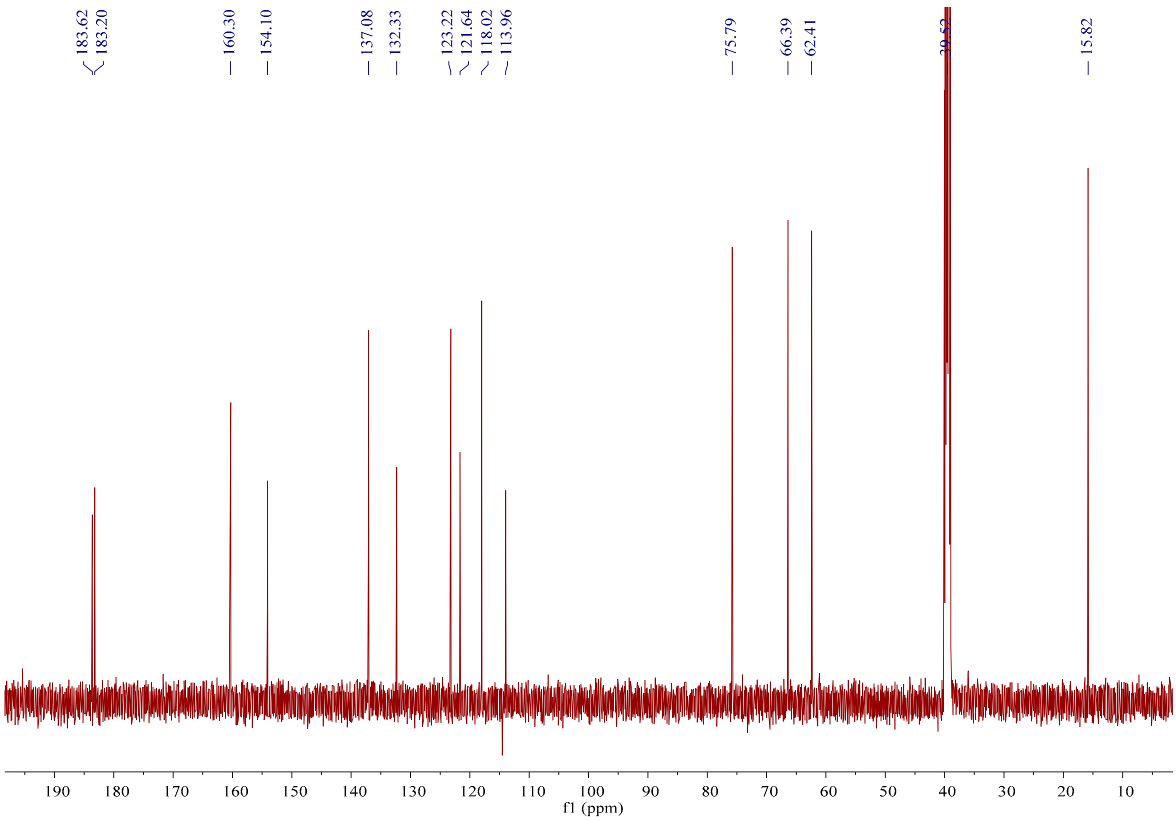


**Figure S21.** $^{13}$C-NMR spectrum of (+)-cryptosporin (**3**) in DMSO-$d_6$.
